# Transmedia Storytelling Research Trends: A Bibliometric Analysis (2020–2025)

**DOI:** 10.12688/f1000research.175171.1

**Published:** 2026-02-04

**Authors:** Karen Patricia Agudelo, Elvira Patricia Flórez Nisperuza, Tobias Parodi-Camano

**Affiliations:** 1 Facultad de Ciencias de la Educación, Universidad de Cordoba, Montería, Cordoba, 230001, Colombia; 2 Ingeniería Industrial, Universidad de Cordoba, Montería, Cordoba, 230001, Colombia

**Keywords:** Transmedia storytelling, bibliometric review, digital education, critical participation, social transformation, interdisciplinary research

## Abstract

**Background:**

Transmedia storytelling has emerged as an essential strategy for connecting content across multiple formats, languages, and platforms, particularly in educational, cultural, and social domains. Despite growing interest, there is still a lack of comprehensive bibliometric syntheses that map global research trends and the intellectual structure of the field.

**Methods:**

A descriptive and retrospective bibliometric study was conducted using the Scopus database to identify scientific production on transmedia storytelling between 2020 and 2025. The study followed the PRISMA methodology under a mixed post-positivist and constructivist approach and applied the ARA framework (Authors, Journals, Contributions) as the main analytical model. Thirty Scopus-indexed journal articles were selected after applying inclusion and exclusion criteria. VOSviewer (2025) was used to generate co-occurrence maps and identify conceptual clusters.

**Results:**

The analysis revealed a predominance of qualitative studies, with strong contributions from Spain, Brazil, Ecuador, Colombia, and Portugal. Spanish and Brazilian authors accounted for more than 60% of the output, confirming an Ibero-American dominance. Thematic analysis identified five major domains: education, cultural heritage, social activism, media innovation, and psychosocial health. Ten main conceptual categories were detected, including transmedia storytelling, adaptation, transmediality, interactive narrative, convergence, higher education, and media convergence. Articles were mainly published in Q1–Q2 Scopus journals in communication, digital education, and cultural studies.

**Conclusions:**

Although still a relatively young field, transmedia storytelling research shows clear interdisciplinary expansion and diversification. The findings highlight the growing academic and social relevance of transmedia storytelling as a driver of social transformation, meaningful learning, and critical participation. Future studies should deepen methodological diversification, promote international collaboration, and strengthen the empirical assessment of social impact, particularly in underrepresented regions of the Global South.

**Abbreviations**
PRISMA: Preferred Reporting Items for Systematic Reviews and Meta-Analyses
XR: Extended Reality
TMS: Transmedia Storytelling
VOS: Visualization of Similarities (VOSviewer)

## Introduction

Transmedia storytelling refers to stories expanded across multiple media platforms that invite audiences to participate as prosumers in building the narrative ( Santin et al., 2024). The integration of technologies such as augmented reality, virtual worlds, and artificial intelligence poses ongoing challenges to contemporary digital communication ( Freire et al., 2023). Within social advertising, digital narratives have become powerful tools to generate new perspectives on social change ( Henao et al., 2025).

Recent years have witnessed growing interest from universities and researchers in studying transmedia storytelling as both an artistic and educational phenomenon ( Piñeiro, 2025). Critical pedagogy emphasizes that in the digital era, citizens must develop media and transmedia literacy to engage meaningfully in networked societies ( Mesquita et al., 2022). This perspective aligns with calls to adapt pedagogical strategies to the realities of digital convergence and interactive learning ( Arias & Del Campo, 2024).

Despite an increase in qualitative and applied studies, a comprehensive bibliometric synthesis of transmedia storytelling remains scarce. Therefore, this study aimed to perform a bibliometric analysis of scientific production related to transmedia storytelling between 2020 and 2025, map global research trends, identify publication patterns, and outline key thematic and methodological tendencies within the field.

## Methods

### Study design

This study adopted a descriptive and retrospective bibliometric design under a mixed post-positivist and constructivist approach. Quantitative indicators were combined with qualitative interpretation to examine patterns of scientific production on transmedia storytelling between 2020 and 2025.

### Data source and search strategy

A descriptive and retrospective bibliometric study was conducted using the Scopus database. Guided by the PRISMA statement Matthew T., et al. (2021), the study combined quantitative indicators with qualitative interpretation, supported by post-positivist and constructivist paradigms.

The ARA framework was applied to classify information into three analytical dimensions:

D1 – Authors: total citations, average citations per year, affiliation, country, h-index ( Table 1).

**Table 1.** Dimension 1 – Authors.

Author(s)	Total Citations	Citations per Year	Year of Publication	Affiliation	Country	h-index
Piñeiro (2025)	399	33	2025	University of Salamanca	Spain	12
Henao et al. (2025)	0	0	2025	Bellas Artes Institution of Valle del Cauca	Colombia	0
Viñaras et al. (2025)	315	26	2025	CEU San Pablo University	Spain	12
Fonts & Durán (2024)	7	4	2024	Polytechnic University of Catalonia	Spain	2
Chamorro (2024)	2	1	2024	University of Granada	Spain	1
De Barros et al. (2024)	6	2	2024	São Paulo State University (UNESP)	Brazil	1
Ortega et al. (2024)	38	5	2024	University of Jaén	Spain	3
Falandes & Renó (2023)	2	1	2023	São Paulo State University (UNESP)	Brazil	1
Leite (2023)	0	0	2023	University of Algarve	Portugal	0
Freire et al. (2023)	46	5	2023	Abat Oliba CEU University	Spain	5
Piñeiro & Crespo (2023)	399	33	2023	University of Salamanca	Spain	12
Sahagún & Fernández (2023)	24	3	2023	Rey Juan Carlos University	Spain	2
García (2022)	1	1	2022	University of Málaga	Spain	1
Loayza (2022)	0	0	2022	Technical University of Loja	Ecuador	0
Guerrero et al. (2022)	420	20	2022	University of Santiago de Compostela	Spain	11
Álvarez & Selva (2022)	47	14	2022	University of Cádiz	Spain	3
Quezada et al. (2022)	8	3	2022	Ismael Pérez Pazmiño Higher Technical Institute	Ecuador	2
Montoya & Páez (2021)	1	0	2021	University of Boyacá	Colombia	0
Andrade & Fonseca (2021)	4	1	2021	Pontifical Catholic University of Ecuador	Ecuador	1
Enríquez & Gómez (2021)	0	0	2021	San Francisco University of Quito	Ecuador	0
Costa & López (2021)	562	61	2021	University of A Coruña	Spain	13
Arrobo & Suing (2021)	18	10	2021	Technical University of Loja	Ecuador	2
Magalhães (2021)	9	8	2021	Catholic University of Portugal	Portugal	2
Vázquez Herrero (2021)	1	1	2021	University of Santiago de Compostela	Spain	1
Ulloa et al. (2020)	7	4	2020	ESPOL Polytechnic University	Ecuador	1
Durán LQ et al. (2020)	17	8	2020	University of Aveiro	Portugal	3
Freire (2020)	21	7	2020	Pontifical Catholic University of São Paulo	Brazil	2
Herrero & De la Maza (2020)	134	12	2020	University Carlos III of Madrid	Spain	7

*Source*: Authors’ own elaboration based on Scopus ( Elsevier; accessed 15 Nov 2025).

D2 – Journals: quartile, indexation, impact factor, and publication scope ( Table 2).

**Table 2.** Dimension 2 – Journals.

Journal title	Main research areas	Quartile	Indexation	Impact factor	Country
Digital Applications in Archaeology and Cultural Heritage	Arts and Humanities – Archaeology	Q1	Scopus	0.532	Netherlands
Communication Research and Practice	Computer Science – Networks, Communications, HCI	Q2	Scopus	0.462	United Kingdom
Risti – Iberian Journal of Information Systems and Technologies	Computer Science – Information Systems and Technologies	Q4	Scopus	0.134	Portugal
Applied Mathematics and Nonlinear Sciences	Computer Science – Applied Mathematics and Modeling	Q4	Scopus	0.250	Poland
Texto Livre	Social Sciences – Communication, Education, Linguistics	Q2	Scopus	0.221	Brazil
Digital Education Review	Social Sciences – Education and Digital Pedagogy	Q2	Scopus	0.392	Spain
Journal on Interactive Systems	Computer Science – Informatics, HCI, Information Systems	Q4	Scopus	0.199	Brazil
Austral Comunicación	Computer Science – Networks, Information Systems	Q3	Scopus	0.130	Argentina
Journal of Science and Technology of the Arts	Arts and Humanities – Conservation, Music, Visual and Performing Arts	Q2	Scopus	0.127	Portugal
Digital Health	Decision Sciences – Health Communication and Informatics	Q1	Scopus	6.838	United Kingdom
Artnodes	Arts and Humanities – Literature, Visual and Performing Arts	Q1	Scopus	0.280	Spain
Multimedia Tools and Applications	Computer Science – Networks, Hardware, Software Architecture	Q1	Scopus	0.777	United States
International Journal of Serious Games	Computer Science – Artificial Intelligence, Computer Graphics, HCI	Q2	Scopus	0.480	Italy
Technology, Knowledge and Learning	Computer Science – Computational Theory, Informatics, HCI	Q1	Scopus	1.210	United States
Observatorio	Computer Science – Network Communications	Q3	Scopus	0.203	Portugal
Tekst, Kniga, Knigoizdaniye	Arts and Humanities – Visual and Performing Arts	Q2	Scopus	0.147	Russian Federation
Digital Humanities Quarterly	Arts and Humanities – Digital Culture	Q2	Scopus	0.270	United States
Historia y Comunicación Social	Communication – History, Journalism, Media Discourse Analysis	Q3	Scopus	0.230	Spain
Revista Latina de Comunicación Social	Communication – Digital Media, Journalism, Cultural Studies	Q2	Scopus	0.350	Spain
Estudos em Comunicação	Communication – Digital Technologies, Media, Society	Q3	Scopus	0.230	Portugal

*Source*: Authors’ own elaboration based on Scopus ( Elsevier; accessed 15 Nov 2025).

D3 – Contributions: themes, methodologies, analytical techniques, and research samples.

**Figure 1.**
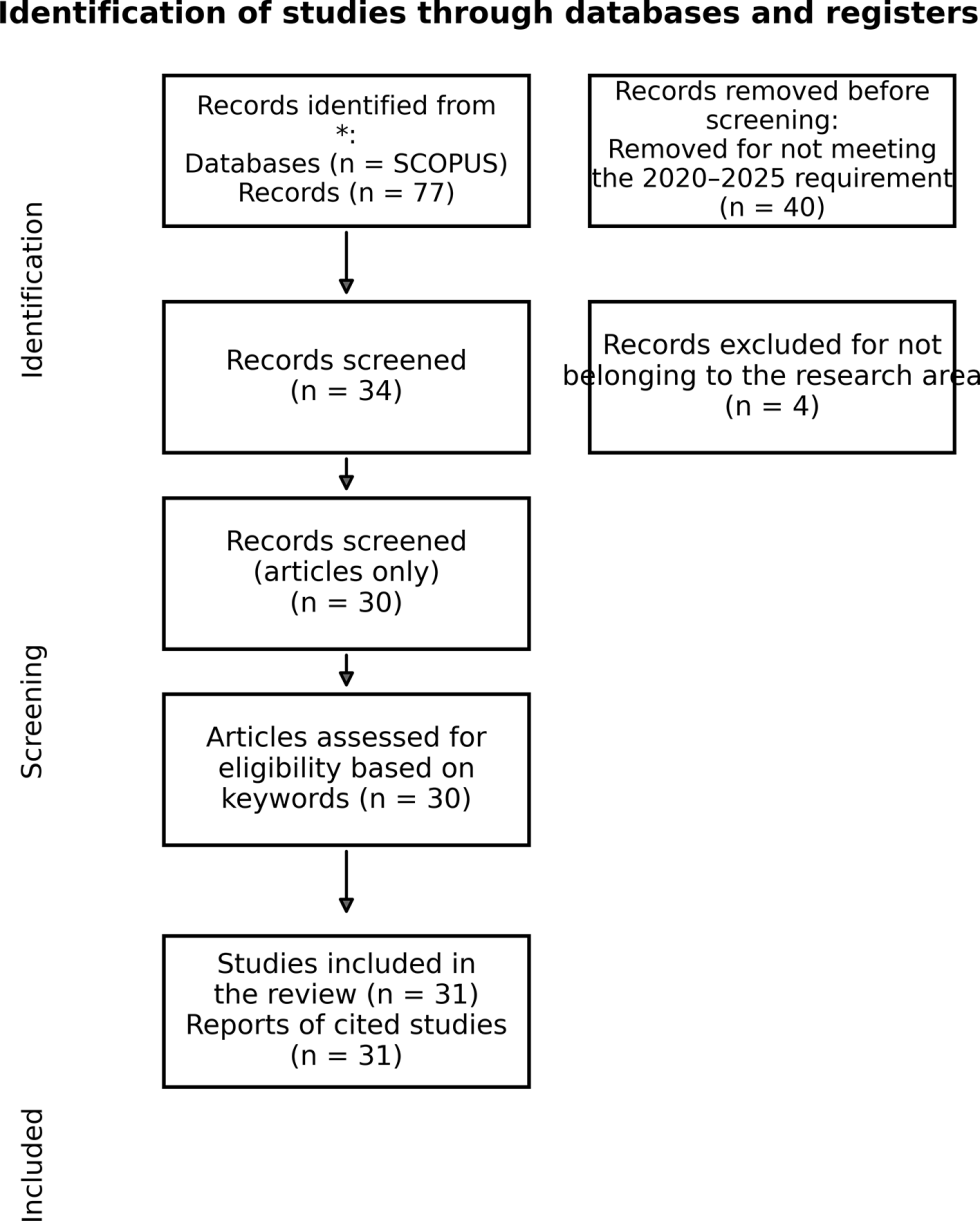
PRISMA flow diagram – adapted from Matthew et al., 2021.

After filtering by publication year (2020–2025), 77 records were retrieved. Following inclusion criteria, 30 peer-reviewed journal articles were retained for analysis ( Figure 1).

TITLE-ABS-KEY (“NARRATIVA TRANSMEDIA” OR “TRANSMEDIA STORYTELLING”) AND PUBYEAR > 2019 AND PUBYEAR < 2026 AND (LIMIT-TO (SUBJAREA, “ENVI”)) AND (LIMIT-TO (DOCTYPE, “ar”)) AND (LIMIT-TO (LANGUAGE, “English”)) AND (LIMIT-TO (EXACTKEYWORD, “Article”)) AND (LIMIT-TO (SRCTYPE, “j”)) AND (LIMIT-TO (EXACTSRCTITLE, “Journal Of Environmental Management”)) AND (LIMIT-TO (OA, “repository”))

The bibliometric visualization was performed with VOSviewer (2025), generating conceptual co-occurrence maps to identify main thematic clusters.

## Results

### Conceptual categories

**Figure 2.**
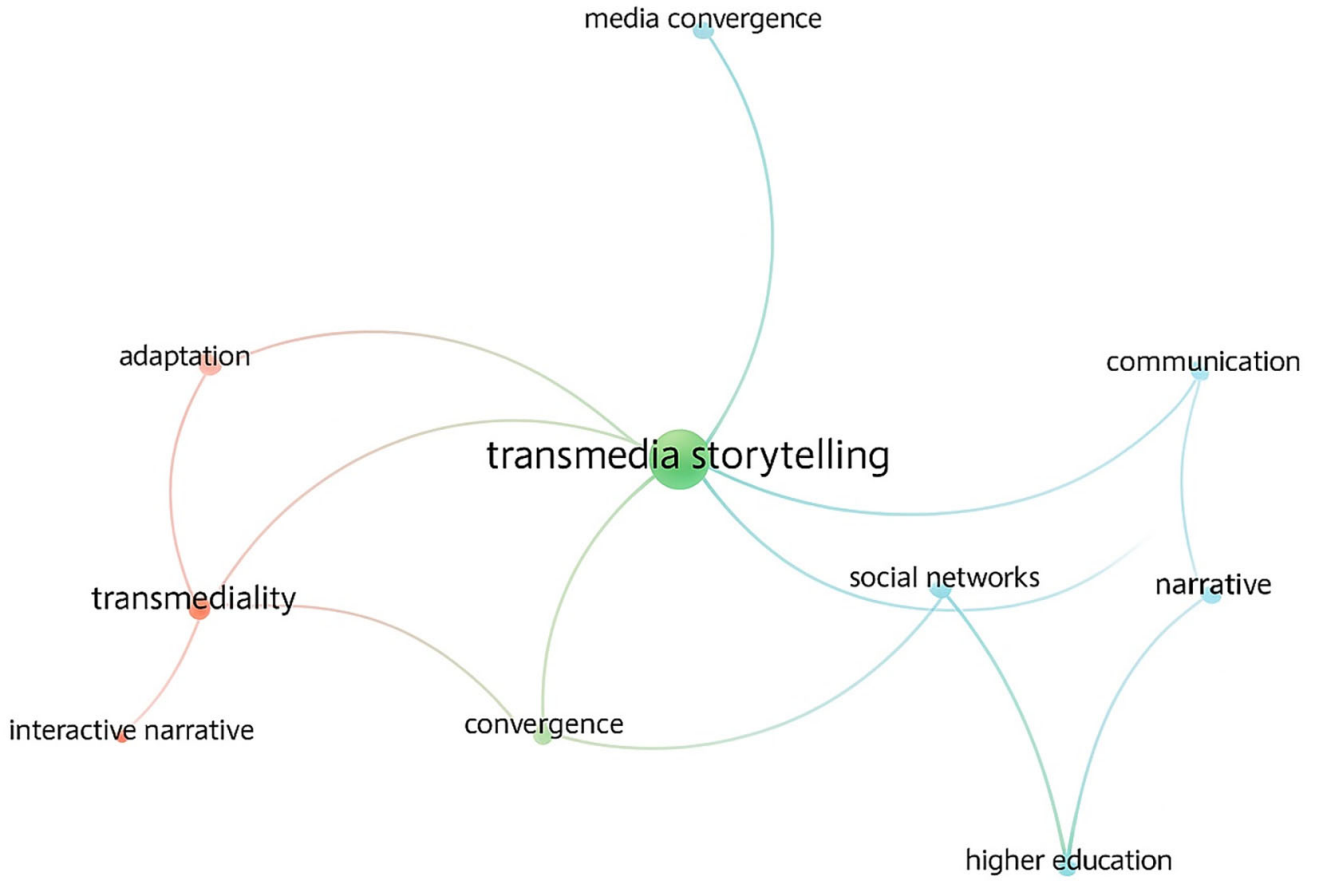
Conceptual categories of the research (Source: VOSviewer, 2025).

The bibliometric analysis using VOSviewer (2025) identified 514 keywords across 30 Scopus-indexed articles. Through co-occurrence analysis (96% confidence level; 4% margin of error), ten major conceptual categories emerged, reflecting the intellectual structure of transmedia storytelling research ( Figure 2).

These categories represent the conceptual backbone of the field, revealing the disciplinary intersections between communication, education, and digital media.

Main Conceptual Categories Identified:

• Transmedia storytelling – the core construct of the field, understood as narrative worlds expanded across multiple media that invite users to co-create meaning ( Ryan, 2022).
• Adaptation – the creative reinterpretation of texts and stories across platforms, enabling narrative transformation rather than mere translation ( Herrero & De la Maza, 2020).
• Transmediality – the ability of stories to develop coherently through diverse media, each contributing unique layers to a broader storyworld ( Ryan, 2022; Canción et al., 2025).
• Communication – a dynamic, participatory process of meaning-making within networked societies ( Requena Santos, 2024).
• Social networks – socio-digital spaces that sustain symbolic interactions and collective creativity ( Requena Santos, 2024).
• Interactive narrative – non-linear storytelling that empowers users through feedback loops and decision-making, producing immersive experiences ( Bullock, Shulman, & Huskey, 2021; Ortega et al., 2024).
• Convergence – the technological and media integration enabling the fluid movement of content across devices ( Freire et al., 2023).
• Higher education – an emerging area using transmedia storytelling to strengthen creativity, media literacy, and participatory learning ( Acevedo, Menese-Camargo, & Da Silva Muñoz, 2024).
• Narrative – a fundamental communicative form central to persuasion and emotional engagement ( Bullock, Shulman, & Huskey, 2021).
• Media convergence – the fusion of traditional and digital media ecosystems, transforming journalism and participatory communication ( Herrero & De la Maza, 2020).

The conceptual network ( Figure 2) shows a progressive evolution from isolated case studies to integrative frameworks linking technological innovation, cultural participation, and educational transformation.

*Dimension 1 – Authors*

The bibliometric analysis revealed a strong concentration of authors from Spain, with emerging contributions from Latin America—particularly Brazil, Ecuador, Colombia, and Portugal. The predominance of institutions such as the University of Salamanca, University of Santiago de Compostela, and Universidade da Coruña confirms Spain’s leading position in this field ( Piñeiro, 2023; Guerrero, 2022; Costa-Sánchez & López-García, 2021).

Spanish and Brazilian authors contribute over 60% of total output, revealing Ibero-American dominance and emerging diversification among Latin-American researchers.

*Dimension 2 – Journals*

Most articles appeared in Q1–Q2 Scopus journals from Europe and Latin America, covering communication, digital education, and cultural studies. Journals such as Digital Health [20], Technology, Knowledge and Learning [24], and Digital Education Review [15] hold the highest impact, reinforcing the interdisciplinary relevance of transmedia storytelling.

*Dimension 3 – Contributions*

Thematic analysis identified five principal domains: education, cultural heritage, social activism, media innovation, and psychosocial health. Representative studies and methodologies are summarized in Table 3.

**Table 3.** Dimension 3 – Contributions.

Theme	Methodology	Analytical technique	Sample/Scope	Key findings	Main conclusions
Integration of Transmedia Storytelling (TMS) and Gamification in Museums through XR Technologies	Qualitative study with phenomenological approach	Semi-structured interviews; thematic analysis based on Heidegger’s quadruple model	27 experts in XR, narrative and museology (Asia, Europe, Canada, U.S.)	Immersive TMS increases participation and transforms museum experiences	Combining TMS and XR redefines visitors as active participants and is recommended for heritage education and entertainment.
Consumption of Fictional Transmedia Narratives among University Students	Quantitative descriptive	Frequency analysis of open-ended responses	230 university students from Spain	Identified 50 fiction titles with 10+ mentions; transmedia consumption is global and genre-conservative	Focus on popular content with limited diversity poses challenges for media literacy.
Relationship between Museums and Generation Z in Transmedia Context	Mixed qualitative and quantitative approach	Focus groups, surveys, thematic analysis	597 young participants from Latin America and Spain	Museums remain symbols of cultural authority but lack shared codes with youth	New transmedia strategies are needed to connect museums with Gen Z through digital culture.
Transmedia Narratives Applied to Historical Memory in the Digital Platform of Chile’s Museum of Memory	Qualitative case study with triangulation	Content analysis and focus group with professionals	Five digital sub-platforms of www.mmdh.cl + expert focus group	Reconstructs memory through multi-story formats and participation	Transmedia memory fosters critical reflection, justice, and digital education for future generations.
Feminist Transmedia Narratives through Podcast Format	Qualitative case study	Critical discourse analysis and case study	Female-led journalistic podcasts in Brazil	Podcasts express female experiences and challenge patriarchal digital logics	Feminist podcasts create spaces of resistance and identity through transmedia strategies.
Transmedia and Artivism as Inclusive Science Communication Strategies	Qualitative descriptive study	Case review and documentary analysis	Artistic and educational projects in Spain	Transmedia and artivism communicate complex science concepts creatively and accessibly	Artivism and transmediality foster co-creation and public understanding of science.
Spherical Narrative Models in Non-Fiction Documentaries Using 360° Technology	Exploratory qualitative research	Theoretical and critical analysis of immersive documentaries	Multiple 360° non-fiction documentaries analyzed from audiovisual poetics approach	Identified features of spherical narrative: immersion, decentralized focus, viewer agency	360° narratives transform non-fiction documentary experience and demand new poetics of sensory immersion.

*Source*: Scopus (2025).

*Development stages*

The temporal evolution of publications revealed three distinct phases:

• Phase 1 (2020–2021): Early exploration of digital journalism and social transmedia storytelling for social awareness.
• Phase 2 (2022–2023): Integration of transmedia storytelling into education through frameworks like INAEP (Investigate, Narrate, Elaborate, Question).
• Phase 3 (2024–2025): Expansion toward digital memory, activism, and affective participation, including museum-based and social media projects.

Publication output peaked in 2021, coinciding with the COVID-19 pandemic, when digital cultural production surged.

*Authors and institutional analysis*

The leading scholars were Costa & López (2021), Piñeiro (2025), and Guerrero (2022), with Spain as the most productive country. Emerging contributions from Latin America (Ecuador, Brazil, Colombia, and Portugal) reflect a diversification of the field but remain under-cited.

*Journals*

Articles appeared mainly in Scopus Q1–Q2 journals such as Digital Education Review, Technology, Knowledge and Learning, and Digital Humanities Quarterly. Publication domains span communication, digital arts, and higher education, evidencing interdisciplinary crossovers ( Digital Education Review, 2023).

## Contributions

Research contributions cluster around five major applications:

1. Cultural heritage: Use of XR and gamified storytelling to enhance museum engagement Canción Z., et al. (2025).
2. Social activism: Narratives addressing gender equality, environmental awareness, and historical memory Loayza J. (2022).
3. Media innovation: Docuwebs and newsgames fostering critical participation Álvarez-Rodríguez V. & Selva-Ruiz D. (2022).
4. Psychosocial health: Transmedia narratives promoting emotional well-being and public health literacy Duran R. et al. (2020).

## Discussion

At this stage, it is essential to provide a reflective discussion aligned with the guiding research question and the main findings of the study. The analysis reveals patterns, tensions, and emerging perspectives within the field of transmedia storytelling, particularly regarding its social, educational, cultural, and communicative applications across Ibero-America. One of the most relevant aspects to emerge from the results is the variety of methodological approaches and the diversity of research objectives that characterize the analyzed studies. This diversity indicates that transmedia storytelling cannot be understood as a homogeneous phenomenon, but rather as a complex, multidimensional framework that integrates technological, narrative, aesthetic, and participatory dimensions ( Guerrero, 2022; Freire et al., 2023).

Such heterogeneity represents a challenge for the development of shared theoretical frameworks, but at the same time opens the way to more transdisciplinary approaches. Regarding the role of audiences, the findings show that transmedia storytelling profoundly transforms reception practices, empowering users as prosumers and co-creators of meaning. Educational projects (García, 2022), activist practices ( Loayza, 2022), and mental health interventions ( Duran et al., 2020) demonstrate that the audience is no longer a passive agent but an active node in narrative construction. Moreover, this participation occurs through digital platforms enriched with emotional, symbolic, and political components, especially in cases addressing memory, gender, and environmental issues ( Chamorro, 2024; Bullock et al., 2021).

From an academic and geographic perspective, despite the evident growth in scientific production, a regional asymmetry persists in which countries such as Spain and Brazil lead transmedia research ( Piñeiro, 2025; Costa-Sánchez & López-García, 2021), while other territories still face significant challenges. The centrality of this topic can be understood in light of research policies and the available digital infrastructure, opening a critical debate on the democratization of knowledge and the need to amplify the voices of the Global South within the transmedia research ecosystem.

Another notable finding concerns the emergence of hybrid and immersive formats—such as docuwebs, newsgames, and XR technologies in museums ( Herrero & De la Maza, 2020; Ortega et al., 2024). These experiences expand the expressive possibilities of transmedia storytelling and enhance its educational potential by promoting experiential, situated, and affective learning ( Duran et al., 2020). In these contexts, transmedia narratives function as interfaces that connect users with complex content through multisensory and interactive experiences, particularly in relation to cultural heritage, environmental awareness, and social memory.

Taken together, this bibliometric analysis (2020–2025) shows that transmedia storytelling has consolidated an interdisciplinary thematic field with a significant presence in countries such as Spain, Brazil, Ecuador, Colombia, and Portugal, and with notable publications in indexed journals addressing communication, education, technology, and digital culture. A key contribution of this study is the identification of thematic approaches that go beyond entertainment-oriented uses: transmedia narratives are increasingly applied to historical memory, environmental activism, health education, political formation, and knowledge appropriation, serving as powerful tools to connect knowledge, create symbolic bonds, and foster active audience participation. This thematic expansion is accompanied by methodological plurality, in which qualitative studies prevail but are complemented by quantitative and experimental approaches, highlighting an ongoing evolution toward social transformation, inclusion, and critical education and reflecting the maturity and adaptability of the field.

This study has several limitations that should be considered when interpreting the findings. First, the analysis is restricted to articles indexed in a single database and within a specific subject area and journal, which may exclude relevant research published in other outlets or in different languages. Second, the relatively small number of documents meeting the inclusion criteria limits the generalizability of the results, even though it allows an in-depth examination of the selected corpus. Third, bibliometric methods provide a structured overview of trends and patterns but do not fully capture the richness of local practices, reception processes, or the long-term social impact of transmedia projects.

Future research could address these limitations by expanding the search to additional databases and subject areas, incorporating multilingual corpora, and including mixed-methods designs that combine bibliometric, qualitative, and audience-based analyses. Comparative studies between regions, especially between the Global North and the Global South, would help clarify how structural inequalities and cultural specificities shape the design, implementation, and reception of transmedia storytelling. Likewise, more empirical work is needed to measure the educational, psychosocial, and civic outcomes of transmedia interventions, particularly in underrepresented contexts such as rural communities, marginalized groups, and low-resource educational settings. It is also essential to investigate the potential of transmedia storytelling to influence public policies, educational innovation, and community development in the digital era.

## Ethical considerations

Not applicable. This study used secondary data derived from previously published, peer-reviewed articles and did not involve human participants or animals.

## Data Availability

The bibliometric dataset was compiled from Scopus-indexed records (2020–2025) following the search strategy described in the Methods section. Due to Scopus licensing restrictions, the raw exported records cannot be redistributed publicly. However, the derived datasets generated by the authors (e.g., author indicators, journal metrics, keyword and thematic coding sheets, and the list of included articles with bibliographic metadata) are available from the corresponding author upon reasonable request. Requests should be sent to Karen Patricia Agudelo Arteaga ( karenagudelo@correo.unicordoba.edu.co). Access will be provided for research and teaching purposes and may require confirmation that the request complies with Scopus/Elsevier terms of use. This bibliometric review was informed by the PRISMA 2020 guidelines for reporting systematic reviews ( Matthew et al., 2021), particularly for documenting the identification, screening, eligibility, and inclusion of studies (see PRISMA flow diagram in Figure 1). No formal PRISMA checklist or flowchart has been deposited in an online repository.
