# Supplementary material for: Transmedia Storytelling Research Trends: A Bibliometric Analysis (2020–2025)
Source: F1000Res. 2026 Feb 4;15:186. [Version 1] doi: 10.12688/f1000research.175171.1 (PMC13191309; doi:10.12688/f1000research.175171.1)
Supplement: Peer review history

# Reviewer report: Reviewer response for version 1

**DOI:** 10.5256/f1000research.193132.r472207

**Published:** 2026-05-20

**Authors:** Adriano Euripedes Medeiros Martins

**Affiliations:** 1 Federal Institute of Education Science and Technology of Triângulo Mineiro, Uberaba, State of Minas Gerais, Brazil

**Peer Review Report: Transmedia Storytelling Research Trends (2020-2025)**

**1. Methodological Aspects and Elements**

 The present study is appropriately characterized as a retrospective-descriptive bibliometric review. The adoption of the ARA framework (Authors, Resources, and Contributions) in articulation with the PRISMA guidelines provides the manuscript with the methodological rigor required for high-impact publications. Additionally, the selection of the Scopus database ensures a high-quality analytical corpus (n=30 articles). As a methodological reflection that may nourish future approaches in this line, it would be of great value to the scientific community to continue documenting the operationalization of eligibility criteria, specifying exact inclusion and exclusion parameters in the screening phases, which maximizes the reproducibility of subsequent replications.

**2. Conceptual Framework and Theoretical Convergence**

 The article accurately identifies a critical paradigmatic shift in transmedia storytelling: the transition from a structural construct centered on entertainment toward a pragmatic tool for social and pedagogical transformation. The use of VOSviewer for mapping descriptors empirically demonstrates that the field is moving toward academic consolidation, hybridizing prosumer theory with approaches from critical pedagogy. The geopolitics of knowledge delineated is notable, verifying a clear Ibero-American hegemony (with Spain and Brazil concentrating >60% of the production). Considering that this concentration evidences a dynamic of epistemic innovation from the "Global South" against the traditional Anglo-Saxon axis, this finding invites a profound analytical reflection on the socio-educational factors that drive this phenomenon in the region, opening an excellent debate for academia.

**3. Critical Analysis of Results**

 The study outstandingly parameterizes three evolutionary phases: an Exploratory Phase (2020-21) as a reaction to the pandemic; an Integration Phase (2022-23) of pedagogical systematization; and an Expansion Phase (2024-25) focused on activism and ethics. The correlation between the peak publication volume in 2021 and the COVID-19 pandemic constitutes a key sociological finding, ratifying that the health crisis served as a catalyst for digital methodologies. This excellent analysis lays a fundamental foundation for future research to evaluate the prospective behavior of this metric trend (stabilization or evolution) as institutions consolidate their transition toward post-pandemic normality.

**4. Limitations and Contributions**

 The manuscript evidences remarkable research probity by explicitly declaring the coverage bias (limited to Scopus) and the sample size restrictions. Its highest impact contribution lies in the synthesis of five applied domains, consolidating itself as a strategic roadmap for future lines of research. Although the statement regarding the exclusivity of Scopus is accurate, this work marks the ideal starting point for the prospective integration of complementary indexed databases (e.g., Web of Science) and regional repositories (e.g., SciELO) in order to cover the full spectrum of scientific production.

**Evaluative Synthesis**

 The research exhibits high internal validity, with a strong structural alignment between the research question and the co-occurrence network analysis. It represents a necessary investigative effort to articulate a convergent interdisciplinary field among technology, art, and education. The technical quality of the article is robust, making it an essential reference for literature on Communication and Educational Technologies in the 2020-2025 quinquennium. By virtue of its methodological soundness, the coherence of the design, and the relevance of its findings, the final decision for this manuscript is Approved.

Is the work clearly and accurately presented and does it cite the current literature?

Yes

If applicable, is the statistical analysis and its interpretation appropriate?

Not applicable

Are all the source data underlying the results available to ensure full reproducibility?

Partly

Is the study design appropriate and is the work technically sound?

Yes

Are the conclusions drawn adequately supported by the results?

Yes

Are sufficient details of methods and analysis provided to allow replication by others?

Yes

Reviewer Expertise:

Educação Profissional e Tecnológica.

I confirm that I have read this submission and believe that I have an appropriate level of expertise to confirm that it is of an acceptable scientific standard.

# Reviewer report: Reviewer response for version 1

**DOI:** 10.5256/f1000research.193132.r477332

**Published:** 2026-05-06

**Authors:** Eduardo David Chalapud Narváez

**Affiliations:** 1 Corporacion Unificada Nacional de Educacion Superior, Bogotá, Bogota, Colombia

The article presents a bibliometric analysis of the scientific output on transmedia storytelling indexed in Scopus between 2020 and 2025. Its aim is to identify global research trends, publication patterns, and the thematic and methodological orientations of the field. The study employs the PRISMA protocol as a methodological guide, the ARA framework (Authors, Journals, Contributions) as an analytical structure, and VOSviewer for generating keyword co-occurrence maps. After applying inclusion and exclusion criteria, the final corpus consisted of 30 articles from journals indexed in Scopus Q1-Q2.

 The results reveal a predominance of Spanish and Brazilian authors (over 60% of total output), five main thematic domains (education, cultural heritage, social activism, media innovation, and psychosocial health), and ten conceptual categories organized around transmedia storytelling as the core concept. The study also identifies three evolutionary phases in the field's production (2020–2021, 2022–2023, 2024–2025) and concludes that transmedia storytelling has consolidated itself as an interdisciplinary field with growing social and educational relevance, albeit with persistent regional asymmetries that limit the visibility of the Global South.

**Criterion 3: Sufficiency of Methodological Details for Replication** **Assessment: Partially met**

 While the article describes the general procedure followed, there are gaps that would make it difficult for another researcher to faithfully replicate the study.

 The search string contains inconsistencies that must be corrected. The string published in the article includes filters such as LIMIT-TO (SUBJAREA, "ENVI") (environmental area) and LIMIT-TO (EXACTSRCTITLE, "Journal Of Environmental Management"), which are entirely unrelated to the topic of transmedia storytelling. The authors are advised to revise the search equation and verify that it produces the initial 77 records, ensuring that all operators and filters are consistent with the subject of the study.

 Additionally, while the article mentions that inclusion and exclusion criteria were applied, these are not clearly listed in the body of the text. For transparency and replicability, the authors should explicitly enumerate these criteria, specifying at minimum the document type, language, subject area, and thematic relevance conditions applied during the screening process.

**Criterion 5: Availability of Source Data for Full Reproducibility** **Assessment: Partially met**

 The figures generated with VOSviewer — particularly Figure 2, which displays the conceptual co-occurrence map — are not legible in the current version of the document. Since these maps constitute the central visual evidence for the findings on conceptual categories, their proper presentation is indispensable. The authors are strongly recommended to export all figures at high resolution (minimum 300 dpi), ensuring that node labels and cluster colors are clearly distinguishable, so that readers can independently interpret and verify the visual results.

 After a careful evaluation of the article *“Research Trends in Transmedia Storytelling: A Bibliometric Analysis (2020–2025)”*, I am pleased to confirm that the manuscript is **approved** in its current form.

 The study demonstrates a solid and coherent methodological design, particularly in the application of bibliometric techniques, which are appropriate for the research objectives. The data sources are relevant and sufficiently robust, and the analytical procedures are clearly described, ensuring transparency and reproducibility.

 Furthermore, the results are presented in a structured and accurate manner, allowing for a clear understanding of the evolution and key trends in transmedia storytelling research. The conclusions are consistent with the evidence provided and contribute meaningfully to the academic field by identifying patterns, gaps, and emerging directions.

 Any suggestions provided during the review process are minor in nature and aimed at enhancing clarity and presentation, rather than addressing fundamental scientific issues.

 For these reasons, I consider the article to be scientifically sound and suitable for indexing.

Is the work clearly and accurately presented and does it cite the current literature?

Yes

If applicable, is the statistical analysis and its interpretation appropriate?

Yes

Are all the source data underlying the results available to ensure full reproducibility?

Yes

Is the study design appropriate and is the work technically sound?

Yes

Are the conclusions drawn adequately supported by the results?

Partly

Are sufficient details of methods and analysis provided to allow replication by others?

Partly

Reviewer Expertise:

Future Studies, Technology Foresight, Digital Futures, Narrative Foresight

I confirm that I have read this submission and believe that I have an appropriate level of expertise to confirm that it is of an acceptable scientific standard.
